# 18F-FDG PET/CT in pure testicular Yolk Sac Tumor in adult

**DOI:** 10.1590/S1677-5538.IBJU.2019.0780

**Published:** 2020-11-18

**Authors:** Francisco Javier García-Gómez, José Luis Villar-Rodríguez, Carmen Beato-Zambrano, Pablo Antonio de la Riva-Pérez, María de la Cinta Calvo-Morón

**Affiliations:** 1 Virgen Macarena University Hospital Department of Nuclear Medicine Seville Spain Department of Nuclear Medicine, Virgen Macarena University Hospital, Seville, Spain; 2 Virgen Macarena University Hospital Department of Pathology Seville Spain Department of Pathology, Virgen Macarena University Hospital, Seville, Spain; 3 Virgen Macarena University Hospital Department of Medical Oncology Seville Spain Department of Medical Oncology, Virgen Macarena University Hospital, Seville, Spain

## CASE PRESENTATION

**Figure 1:**
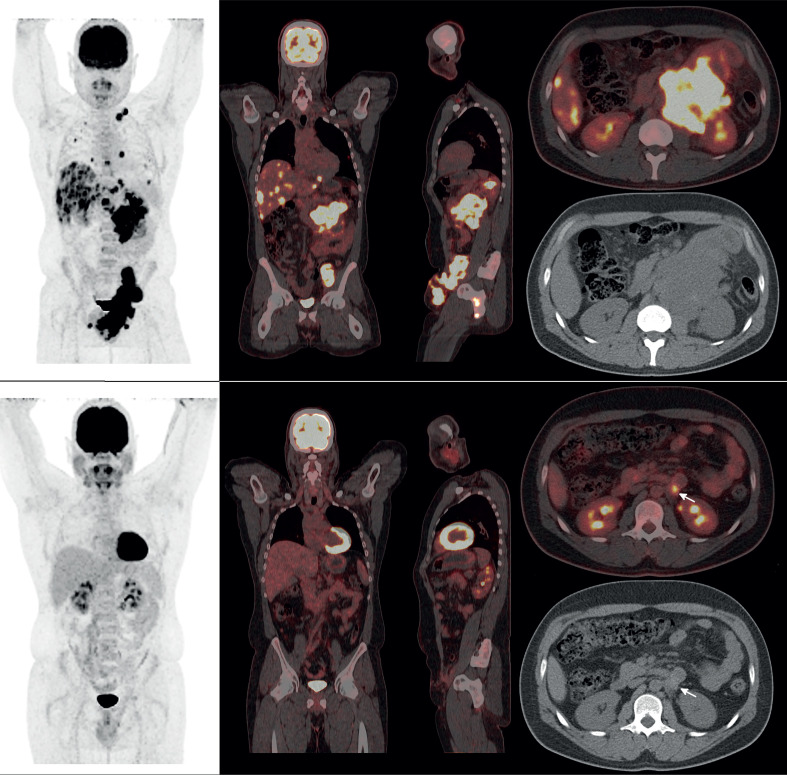
Upper panel: Postoperative 18F-FDG PET/CT scan (MIP and fused coronal, sagittal and axial slices) revealed extensive scrotal-inguinal, pelvic, mesenteric, retroperitoneal, hepatic, pulmonary, supradiaphragmatic and skeletal dissemination, with high metabolic-rate (SUVmax: 22.7). Lower panel: Post-treatment 18F-FDG PET/CT (MIP and fused coronal, sagittal and axial slices) revealed an almost complete metabolic remission after four cycles, with the persistence of retroperitoneal minimal residual disease (arrows, SUVmax 6.1) that are candidates for surgical or radiotherapy treatment.

We report a 35-year-old-male orchidectomized because of a painless left testicular mass. Reactive left inguinal lymphadenopathies were visualized by post-operative ultrasound while findings compatible with orchiepididymitis with necrosis, testicular abscess or underlying neoformation process were found by contrast-enhanced CT. The pathological diagnosis of the surgical piece allowed the diagnosis of pure testicular yolk sac tumor (YST). While YST is a part of mixed non-seminomatous germ neoplasms in up to 79% of cases in the post-pubertal age (1), the pure form observed in this case is referred in only 2.4% of adult patients (2). YST usually behaves as a less aggressive tumor than embryonic carcinoma, so the diagnosis of extensively advanced disease is extremely rare therefore (1). Metastasis usually occurs only through lymphatic spread in adults as opposed to lymphatic and hematogenous spread in prepubescent patients. Serum markers and 18F-FDG PET/CT may assist with the achievement of a differential diagnosis and provide an indicator of patient prognosis in this context. Serum AFP was 5200ng/mL. Additionally, the postoperative 18F-FDG PET/CT scan revealed extensive dissemination with high metabolic-rate (Figure 1, upper panel). Postoperative chemotherapy with three to four cycles of PEB (cisplatin, etoposide and bleomycin) regimen offers a chance for cure in extensively advanced patients (3). In our case, post––treatment 18F-FDG PET/CT revealed an almost complete metabolic remission after four cycles (Figure 1, lower panel).

**Figure 2:**
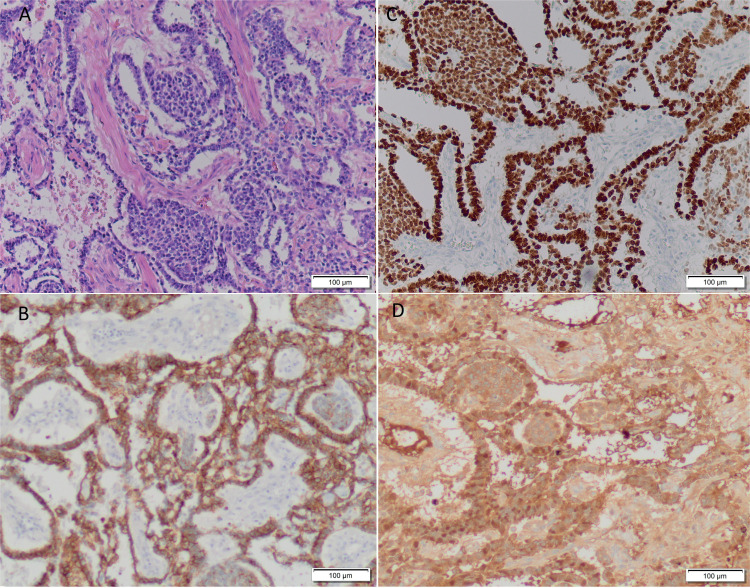
The typical perivascular pattern with the characteristic Schiller-Duval bodies was predominantly observed in the conserved areas (A, Hematoxylin and Eosin stain at x10 magnification). Immunohistochemistry showed strong and generalized expression of pancytokeratin (B, at x10 magnification), SALL4 (C, at x10 magnification) and AFP (D, at x10 magnification). CDX2 was focally expressed (<10% of the neoplasm), while OCT4, CD10, CD30, FAP or CD117 were not expressed, Ki67 index was 90%.

Diagnosis of pure YST, post-pubertal type, should be made only after meticulous microscopic examination rules out other germ cell components (3). In our case, pathological results (Figure 2) revealed a large neoplasm with extensive areas of necrosis that completely affected the testicle, the epididymis, and the spermatic cord, with involvement of surgical resection edges. The typical perivascular pattern with the characteristic Schiller–Duval bodies was predominantly observed in the conserved areas (Figure 2A). These Schiller-Duval bodies are a hallmark of YST seen in 50%-75% of cases (3). YST that present with only one histological pattern are extremely rare (3).
